# CMRD-Net: a deep learning-based *Cnaphalocrocis medinalis* damage symptom rotated detection framework for in-field survey

**DOI:** 10.3389/fpls.2023.1180716

**Published:** 2023-06-08

**Authors:** Tianjiao Chen, Rujing Wang, Jianming Du, Hongbo Chen, Jie Zhang, Wei Dong, Meng Zhang

**Affiliations:** ^1^ Institute of Intelligent Machines, Hefei Institutes of Physical Science, Chinese Academy of Sciences, Hefei, China; ^2^ Science Island Branch, University of Science and Technology of China, Hefei, China; ^3^ Institutes of Physical Science and Information Technology, Anhui University, Hefei, China; ^4^ Agricultural Economy and Information Research Institute, Anhui Academy of Agricultural Sciences, Hefei, China; ^5^ Jingxian Plant Protection Station, Jingxian Plantation Technology Extension Center, Xuancheng, China

**Keywords:** *Cnaphalocrocis medinalis*, damage symptom, deep learning, rotated object detection, horizontal object detection

## Abstract

The damage symptoms of *Cnaphalocrocis medinalis (C.medinalis)* is an important evaluation index for pest prevention and control. However, due to various shapes, arbitrary-oriented directions and heavy overlaps of *C.medinalis* damage symptoms under complex field conditions, generic object detection methods based on horizontal bounding box cannot achieve satisfactory results. To address this problem, we develop a *Cnaphalocrocis medinalis* damage symptom rotated detection framework called CMRD-Net. It mainly consists of a Horizontal-to-Rotated region proposal network (H2R-RPN) and a Rotated-to-Rotated region convolutional neural network (R2R-RCNN). First, the H2R-RPN is utilized to extract rotated region proposals, combined with adaptive positive sample selection that solves the hard definition of positive samples caused by oriented instances. Second, the R2R-RCNN performs feature alignment based on rotated proposals, and exploits oriented-aligned features to detect the damage symptoms. The experimental results on our constructed dataset show that our proposed method outperforms those state-of-the-art rotated object detection algorithms achieving 73.7% average precision (AP). Additionally, the results demonstrate that our method is more suitable than horizontal detection methods for in-field survey of *C.medinalis*.

## 1 Introduction

**Figure 1:**
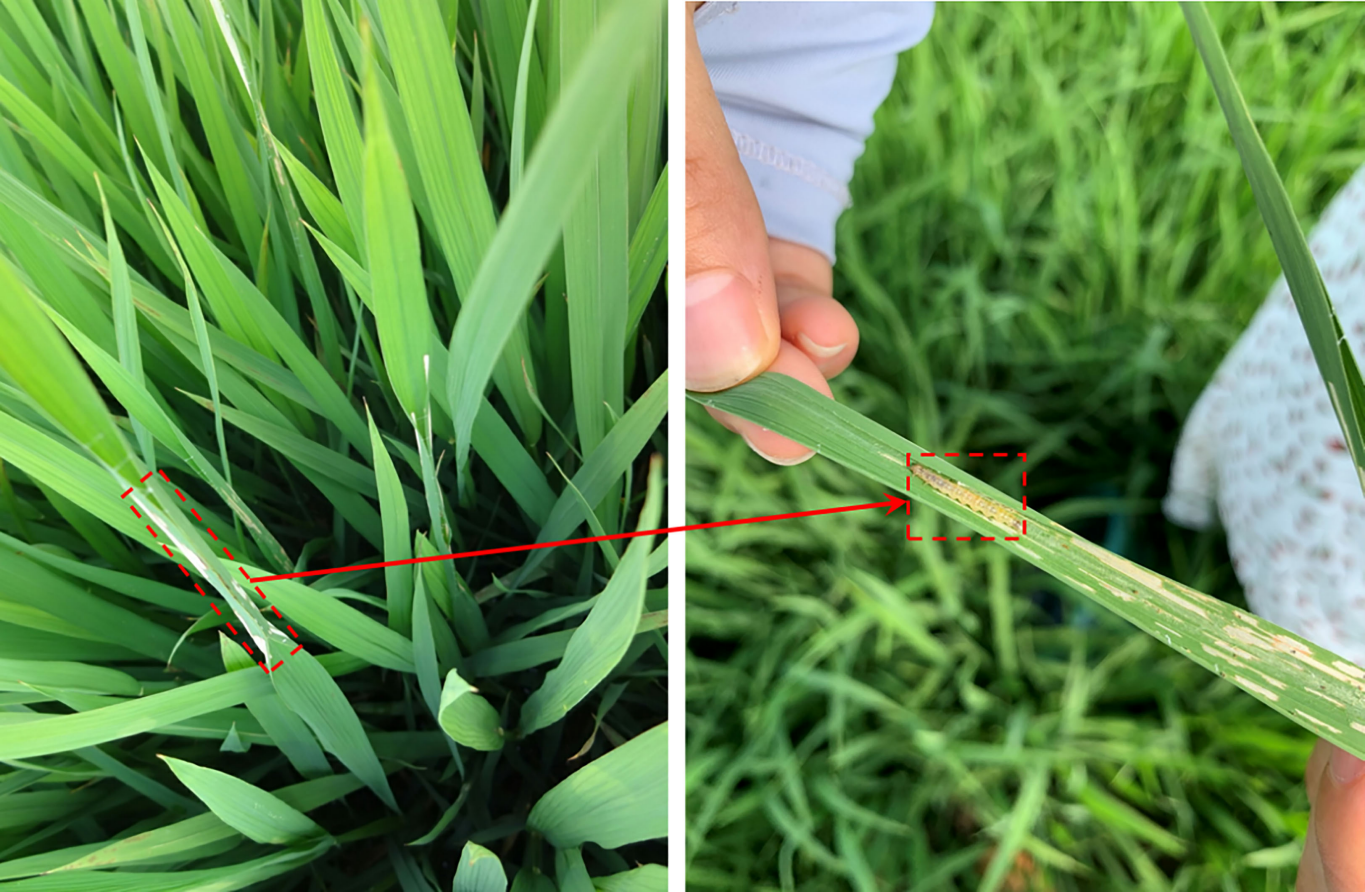
Examples of *C.medinalis* and its damage symptoms. The dashed box in the left panel indicates the typical damage symptom and the arrow points to the pest hiding in the rolled leaf.

*Cnaphalocrocis medinalis(C.medinalis)* always damages rice and results in yield reduction (Heong et al., 1993; Wan et al., 2015). The larvae spit out the silk and roll up the rice leaves from the edges to the center, feeding on the leaf flesh and remaining white damage symptoms, as shown in **Figure 1**. For the pests are concealed in the rolled leaves, plant protection staff have to visually inspect and record the number of rolled leaves with white damage symptoms from the sampled rice clusters, which is inefficient and labor-intensive. With the obvious shortage of plant protection workers and technical strength, automatic monitoring and intelligent investigation of pests and diseases show important research significance. With the development of machine learning technology, extensive studies have been conducted on object detection and recognition methods for rice disease and pest damage symptom images without complex backgrounds. However, the diversity and complexity of in-field scenes make it difficult to determine an optimal feature to solve symptom detection and recognition by traditional machine learning methods (Phadikar et al., 2013; Xiao et al., 2018; Sahu and Pandey, 2023).

**Figure 2:**
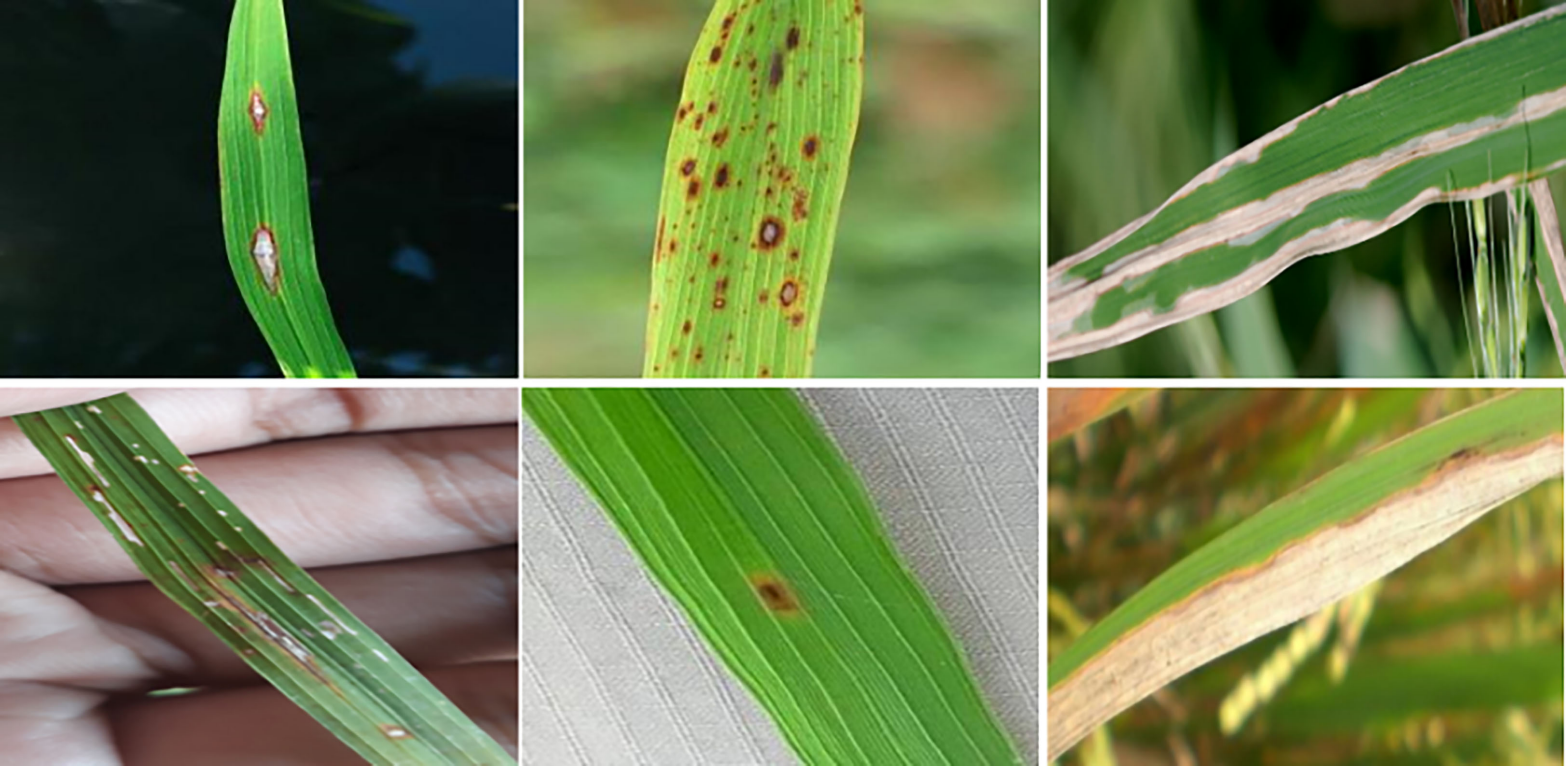
Samples focused on a single leaf with simple backgrounds.

Deep learning is an evolution of machine learning techniques. It can adaptively learn complex and abstract features without human involvement. It has also been widely used in agriculture in recent years, including rice disease or pest damage symptom identification (Sethy et al., 2020; Krishnamoorthy et al., 2021; Wang et al., 2021; Dey et al., 2022). Although these methods made use of deep learning methods to classify rice diseases or pest damage symptoms, they dealt with images focused on a single leaf with simple backgrounds, as shown in **Figure 2**. Deep learning models were trained for image classification using disease data collected in the field (Lu et al., 2017; Rahman et al., 2020). Real-time diagnosis systems were developed for rice disease identification under wild field conditions, combining deep learning techniques with the Internet of Things (IoT) and providing feedback on rice disease categories in response to input images (Temniranrat et al., 2021; Debnath and Saha, 2022; Yang et al., 2022). All of the above methods are used to identify symptom categories. At the same time, the actual demand for pest and disease investigation requires estimating the number of damaged leaves or the area of damaged regions from multiple rice plants or clusters. The precise location of disease or pest damage symptoms under complex field conditions can provide more detailed information to facilitate accurate analyses of the occurrence trend of pest and disease outbreaks.

Since most damage symptoms show discontinuity, pixel-level segmentation methods based on deep learning require heavy labeling work in complex field scenarios. Existing methods for locating symptom areas in the field mostly rely on instance-level bounding box detection. Several works (Zhou et al., 2019; Li et al., 2020) trained an image detection model based on the Faster-rcnn algorithm (Ren et al., 2017) with rice disease datasets collected in the field. The feature pyramid structure was improved on the conventional RetinaNet (Lin et al., 2017b) model and applied to the automatic detection of two pest damage symptoms in the rice canopy (Yao et al., 2020). YoloX (Ge et al., 2021) was adopted in the detection stage to locate the diseased spot areas for subsequent disease classification (Pan et al., 2023). Studies on rice disease or pest damage symptom detection in natural scenes are limited, and prevalent studies mainly employ generic object detection methods based on horizontal bounding boxes (HBBs), which include one-stage and two-stage algorithms. One-stage algorithms usually regress the category and location directly on the feature vectors of key points, such as (Law and Deng, 2018; Duan et al., 2019; Tian et al., 2019; Zhu et al., 2020; Chen et al., 2021; Dai et al., 2021). The rcnn series of algorithms are representatives of two-stage methods, such as typical Faster-rcnn (Ren et al., 2017) and others (Cai and Vasconcelos, 2018; Pang et al., 2019; He et al., 2017; Wu et al., 2020), which select promising proposals based on feature point vectors and map them to feature maps for further fine-tuning. Techniques in object detection continue to be updated, which also promotes the development of deep learning for agricultural disease and pest damage symptom detection.

Due to the oriented and densely-distributed properties of objects in the field, one horizontal bounding box often contains several instances. Comparatively, rotated bounding boxes are more practical to precisely characterize damage symptom areas. However, the convolution kernels in the backbone network of rotated detectors are still horizontal with fixed size. The receptive field associated with the feature vectors of the key points does not carry object shape and tilt information, leading to feature misalignment within one-stage rotated detection algorithms (Liu et al., 2017; Guo et al., 2021; Han et al., 2021; Yang et al., 2021; Li et al., 2022). Although feature maps are refined in many of the above works, one feature point vector remains inadequate to represent a skewed and slender object. Proposals derived from feature point vectors in two-stage methods are projected onto feature maps, and feature-aligned regions will be fetched for final regression and classification. Many rotated anchors were designed in the region proposal network (RPN) to learn targets (Ma et al., 2018). Since the work set rotated anchors with different sizes, aspect ratios, and angles, it will increase a large amount of computation and memory occupation. The Rotated Faster-rcnn method in the MMRotate (Zhou et al., 2022) obtained horizontal proposals in the RPN stage and added an orientation dimension in the region convolutional neural network (RCNN) which is improved on the Faster-rcnn method (Ren et al., 2017). Horizontal proposals were also used in several other rotated detectors (Jiang et al., 2017; Yang et al., 2019; Xu et al., 2021). However, the feature areas corresponding to horizontal proposals will contain much redundant and ambiguous information, which is not conducive to the following detection. Ding et al. (2019) performed feature alignment again using rotated proposals learned in the first RCNN phase based on horizontal proposals, making the parameter number and the computation amount increase.

**Figure 3:**
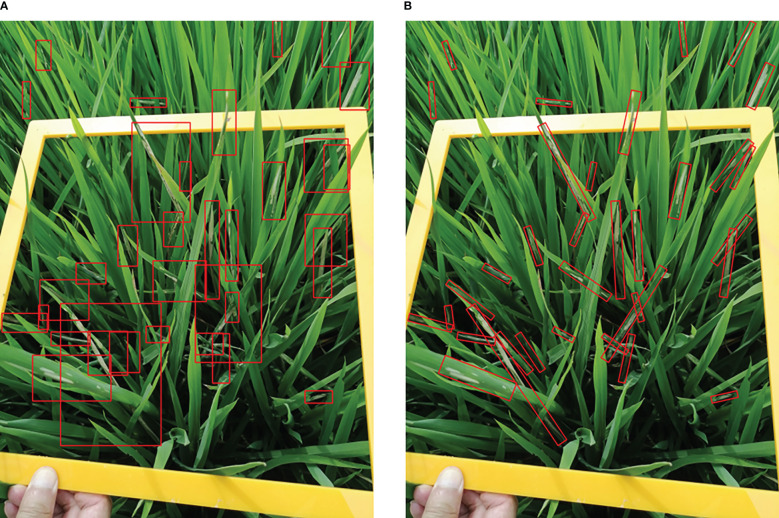
Object representation. **(A)** *C.medinalis* damage symptoms are represented by horizontal bounding boxes and **(B)** rotated bounding boxes.

**Figure 4:**
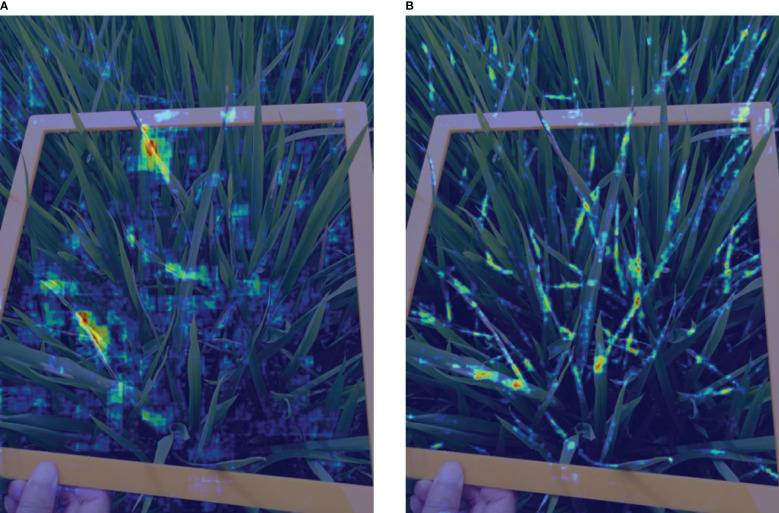
Feature maps. **(A)**the feature map of horizontal detector and **(B)**rotated detector.

Recent detection methods have a couple of limitations in pest damage detection: (1) Horizontal bounding boxes ignore orientation information which is not conducive to expressing the morphology of the damage symptoms precisely. One horizontal bounding box often contains several instances leading to ambiguous features, as shown in **Figure 3**. (2) The horizontal detectors are not able to extract discriminative features for inclined and crossed object detection, as shown in **Figure 4**. Therefore, we propose a two-stage rotated detection framework CMRD-Net for detecting *C.medinalis* damage symptoms. CMRD-Net comprises a Horizontal-to-Rotated region proposal network(H2R-RPN) and a Rotated-to-Rotated region convolutional neural network (R2R-RCNN). First, the H2R-RPN converts horizontal anchors to rotated proposals, and uses adaptive dynamic selection of positive samples (Zhang et al., 2020) to alleviate the hard matching of horizontal anchors due to arbitrary orientation and slender shape of the damage symptoms. Second, the R2R-RCNN performs rotated feature alignment to recalibrate rotated proposals for ultimate pest damage symptom detection. Finally, we establish a rotated bounding box annotation dataset (CMRD) with roLabelImg software for *C.medinalis* damage symptoms and built the horizontal bounding box annotation dataset(CMHD) by fetching horizontal circumscribed rectangles within the CMRD dataset. Extensive experiments demonstrate that our method outperforms other state-of-the-art rotated algorithms. We also verify that the proposed CMRD-Net is more suitable than horizontal detectors for detecting *C.medinalis* damage symptoms.

The main contributions of our work can be summarized as follows:1) The CMRD-NET network is proposed based on rotated bounding boxes to detect *C.medinalis* damage symptoms. CMRD-NET utilizes a Horizontal-to-Rotated proposal strategy and rotated feature alignment to improve accuracy and efficiency. 2) For the detection of *C.medinalis* damage symptoms, extensive experiments have been conducted to demonstrate that our rotated detector is more practical than horizontal detectors, which can locate inclined damage symptoms precisely and enhance the visualization ability of detection results. 3) We construct the CMRD dataset based on rotated bounding box annotation and the corresponding horizontal circumscribed rectangles-based dataset CMHD to demonstrate the effectiveness of our proposed framework, which also provide a richer benchmark for the *C.medinalis* damage symptom detection task.

## 2 Materials and methods

### 2.1 Datasets

#### 2.1.1 Image acquisition

The images in the presented dataset were collected by experts from plant protection stations in 24 cities and counties in China over three years, from 2019-2021, with restrictions on angles and heights during photography. The dataset includes 3900 images with different rice fertility periods and field types. It provides a valuable data resource for *C.medinalis* field surveys and occurrence regularity studies.

#### 2.1.2 Image annotation

**Figure 5:**
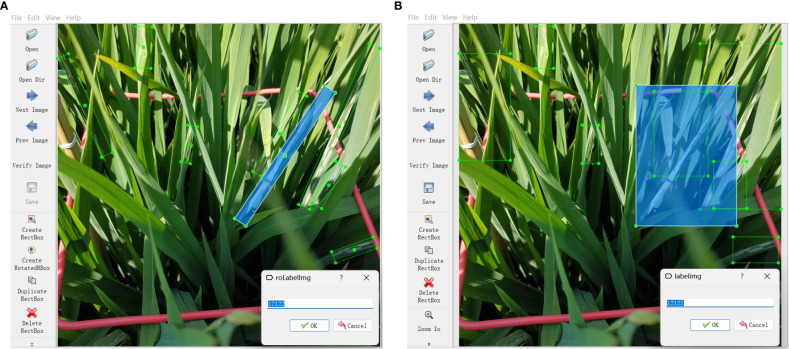
Annotation software. **(A)** Rotated bounding box labeling software roLabelImg and **(B)** horizontal bounding box labeling software labelImg.

The images were annotated with the rotated bounding box labeling software roLabelImg (https://github.com/cgvict/roLabelImg, **Figure 5A**) rather than the horizontal bounding box labeling software labelImg (https://github.com/tzutalin/labelImg,**Figure 5B**). They were saved in XML files after annotation. A vector (x,y,w,h,angle) is used to represent a rotated bounding box closely surrounding a *C.medinalis* damage symptom, where (x,y) refers to its center point coordinates, (w,h) denotes the width and height of the bounding box. The parameter angle represents the radian between the w and x axis with the cycle period of π . Experts from Jingxian Plant Protection Station and Anhui Academy of Agricultural Science perform image labeling in collaboration. The dataset is named *Cnaphalocrocis medinalis* Rotated Dataset (CMRD).

To fairly compare the performance of horizontal detectors and rotated detectors, horizontal circumscribed rectangles are extracted for all rotated bounding boxes in the CMRD dataset, resulting in a *C.medinalis* horizontal annotation dataset called CMHD. Objects are defined as $\left(x_{1},y_{1},x_{2},y_{2}\right)$ in the CMHD dataset, where $\left(x_{1},y_{1}\right)$ represents the top-left vertex coordinates of the horizontal rectangular box and $\left(x_{2},y_{2}\right)$ represents the bottom-right vertex coordinates.

#### 2.1.3 Properties of the CMRD dataset

**Figure 6:**
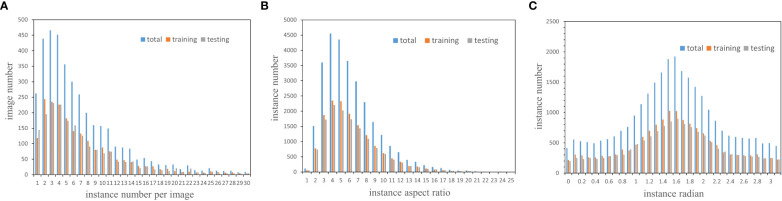
Distribution of the CMRD dataset. **(A)** shows the image number associated with the instance number, **(B, C)** calculate the instance number by aspect ratio and radian. Blue, red and gray denote the distribution of the total dataset, the training set and the testing set.

**Figure 7:**
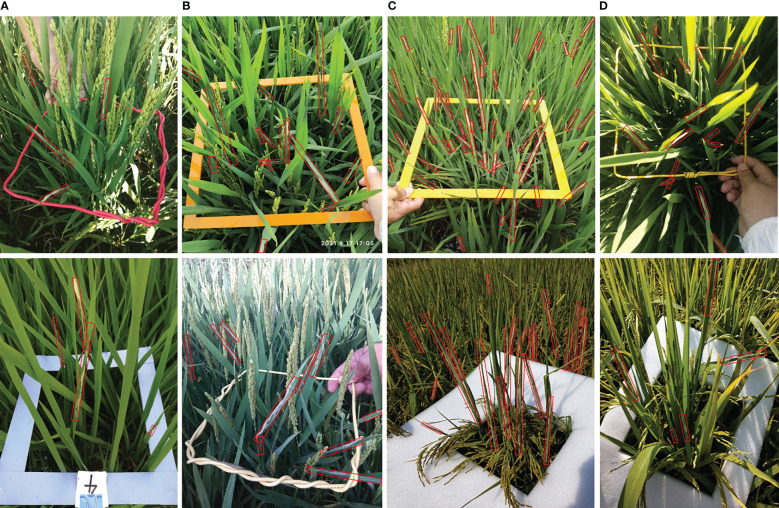
Examples of test subsets. The columns from left to right display two samples of the test subsets **(A)** “sparse”, **(B)** “medium”, **(C)** “dense” and **(D)** “sunlight”.

The dataset was established with different rice field types and fertility periods in different regions, resulting in a diverse and complex dataset. We randomly divided the 3900 images into 2000 training images and 1900 testing images to ensure that the testing set included as many different in-field scenes as possible. The number of instances in each image was counted and the statistical result is shown in **Figure 6A**. In order to compare and analyze the model performance more comprehensively, up to 120 images with the number of instances in the interval 1-9, 10-19, 20 and above were randomly selected from the testing set and named “sparse”, “medium” and “dense”, respectively. 120 images with the influence of sunlight illumination were manually chosen and named “sunlight”. The statistics of all data sets are listed in **Table 1**. The aspect ratio of *C.medinalis* damage symptoms in the field varies widely, and instances incline randomly with the growth direction of the leaves. We counted the number of corresponding instances by aspect ratio and angle, respectively, as shown in **Figures 6B, C**. More than 50% of the instances have an aspect ratio greater than 5, and the instances are randomly oriented with different angles. **Figure 7** lists some samples in different test subsets.

**Table 1:** Statistics on CMRD and its subsets. “average” denotes the average number of instances per image in the corresponding dataset.

Datasets	images	instances	average
total	3900	28964	7.43
training	2000	15117	7.56
testing	1900	13847	7.29
sparse	120	526	4.38
medium	120	1529	12.74
dense	113	2885	25.53
sunlight	120	977	8.14

### 2.2 Methods

#### 2.2.1 Overview of the proposed method

**Figure 8:**
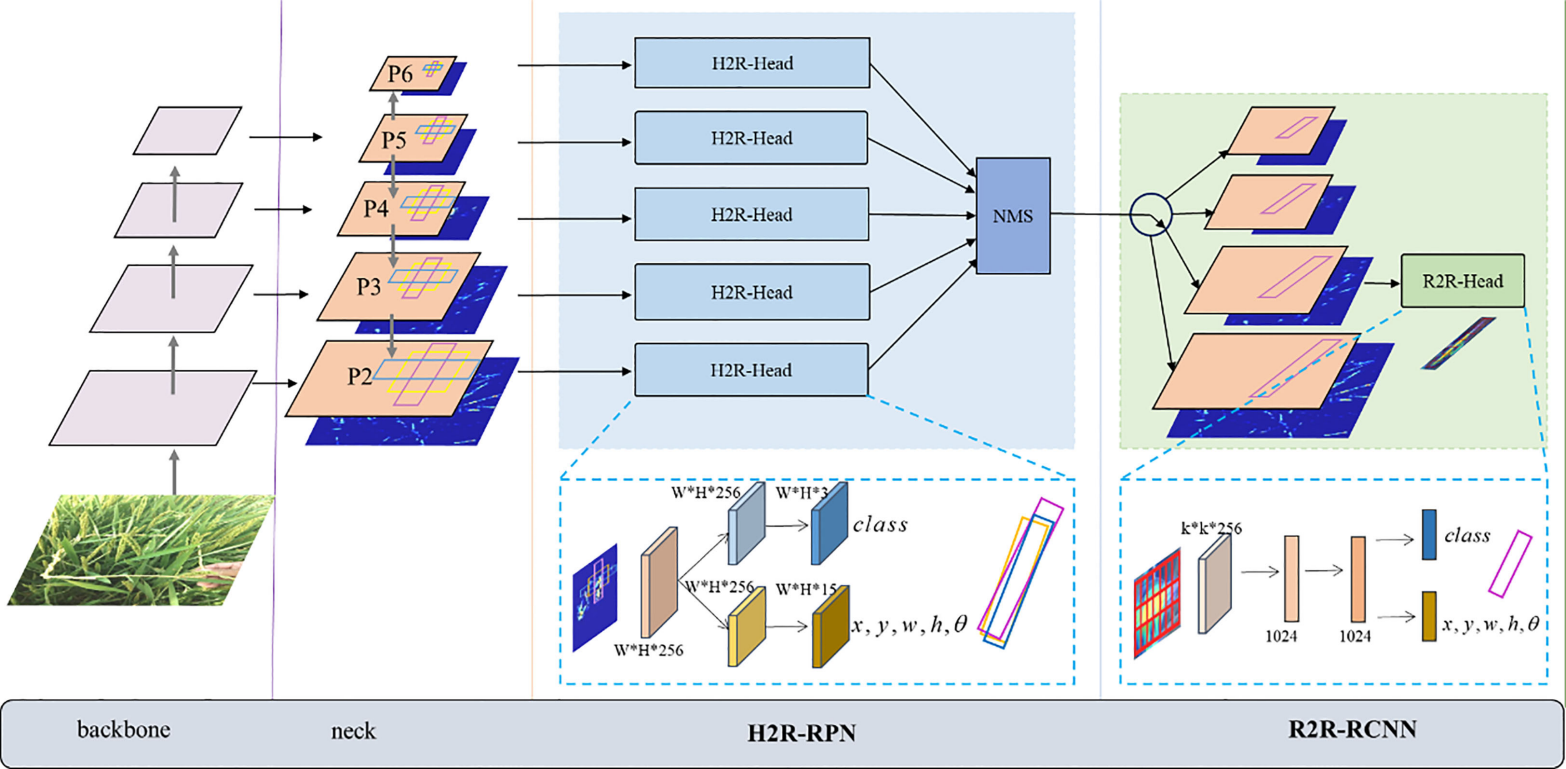
The overall architecture of CMRD-NET. The H2R-RPN module uses horizontal anchors to generate rotated proposals. The rotated proposals are refined in the R2R-RCNN stage based on the oriented-aligned feature regions.

**Figure 9:**
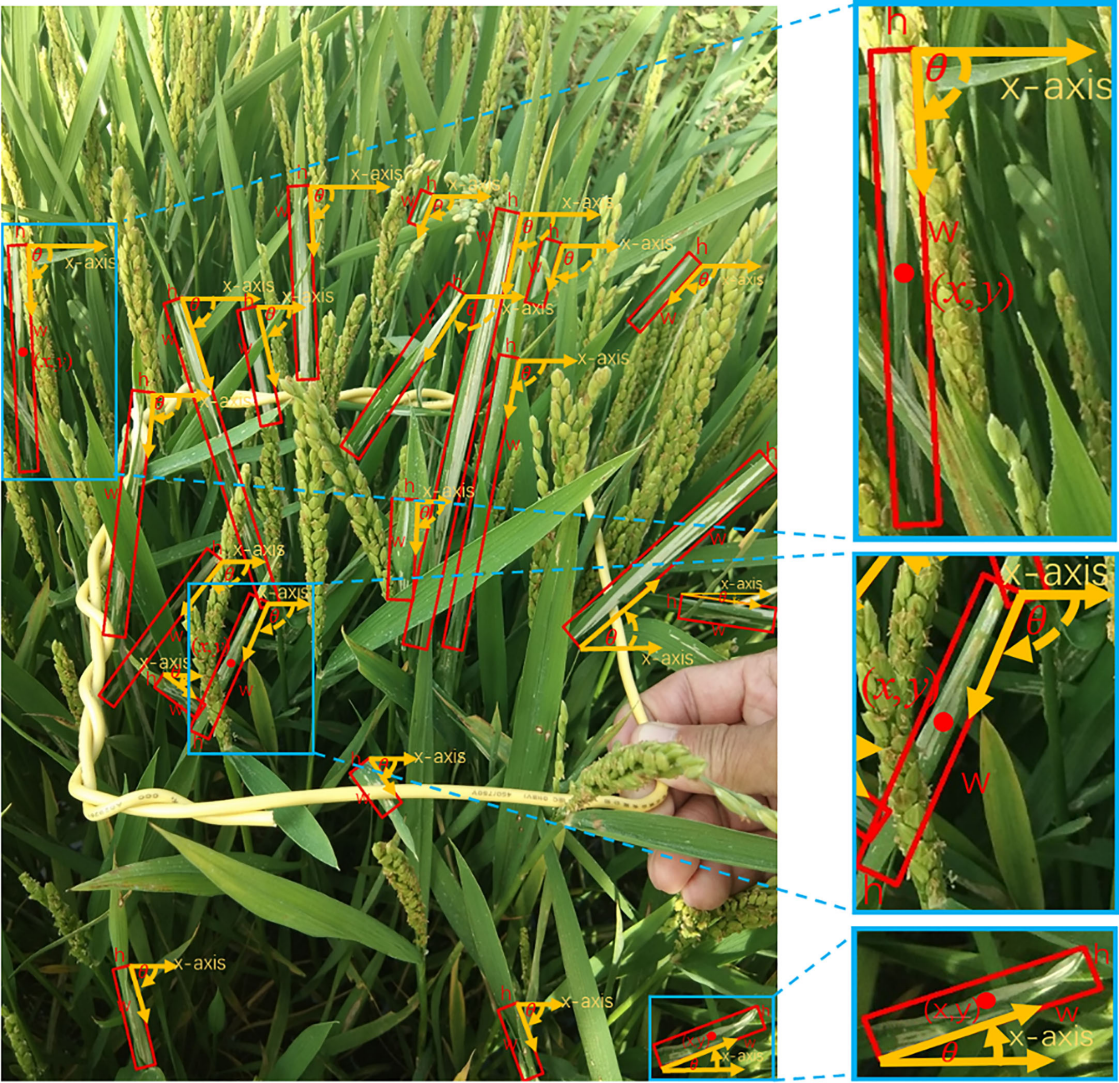
Definition of rotated bounding boxes. (x,y) denotes the center point coordinates, w is the long edge, h is the short edge, and the angle θ is defined by the long edge w and the x-axis in the range of [−π/4,3π/4) . The clockwise angles are positive and the counterclockwise angles are negative.

*C.medinalis* damage symptoms have elongated shapes with large aspect ratios and arbitrary tilt directions, making aligned features essential for detection. We present a rotated detection network CMRD-Net for detecting in-field *C.medinalis* damage symptoms, which outputs oriented bounding boxes that can precisely express the morphology of *C.medinalis* damage symptoms. The overall architecture of CMRD-NET is shown in **Figure 8**. The architecture adopts common settings for the backbone and neck network with ResNet (He et al., 2016) and Feature Pyramid Network(FPN) (Lin et al., 2017a) to extract multi-scale features. The first stage is the H2R-RPN, which sets three horizontal anchors on one feature point to provide rotated proposals to instruct the following module where to look. The adaptive positive sample selection method (Zhang et al., 2020) is introduced to mitigate hard matching between horizontal anchors and inclined instances. The following stage is the R2R-RCNN, which aims at oriented feature alignment and proposal refinement. Features aligned with rotated proposals are extracted from feature maps and used to refine the rotated proposals after down-sampling. In CMRD-Net, we use the long-edge definition method (Ma et al., 2018) with five parameters (x,y,w,h,θ) to represent an oriented object. (x,y) denotes the center point coordinates, w is the long edge, h is the short edge, and the angle θ is defined by the long edge w and the x-axis in the range of [−π/4,3π/4) , as shown in **Figure 9**.

#### 2.2.2 Horizontal-to-Rotated RPN

To reduce the computational burden of numerous rotated anchors, the H2R-RPN assigns three horizontal anchors at each feature point position in different layers of the feature pyramid network P2-P6 with aspect ratios of (0.5,1,2) . As the feature level deepens, the anchor areas are set as $\left(32^{2},64^{2},128^{2},256^{2},512^{2}\right)$ . For a size of W*H*256 feature map in **Figure 8**, it has W*H spatial feature point vectors with 256 channels. The H2R-RPN predicts three rotated proposals with each feature point vector. The classification branch outputs W*H*3(W*H*3*1) scores that provide the probability of object or not object and the regression layer outputs W*H*15(W*H*3*5) encoding five parameters (x,y,w,h,θ) of a predicted rotated bounding box, where the coordinate (x,y,w,h,θ) means the center point, long edge, short edge and angle. As horizontal bounding boxes cover more ineffective areas than rotated bounding boxes, the H2R-RPN learns rotated proposals directly based on predefined anchors. Then up to 2000 rotated proposals in each feature pyramid layer with the highest confidence scores are selected and performed with non-maximum suppression (NMS) (Neubeck and Van Gool, 2006) to remove duplicate detection boxes. Finally, after aggregating proposals from P2-P6 layers, some rotated proposals are collected for subsequent fine-tuning based on confidence score sorting.

**Figure 10:**
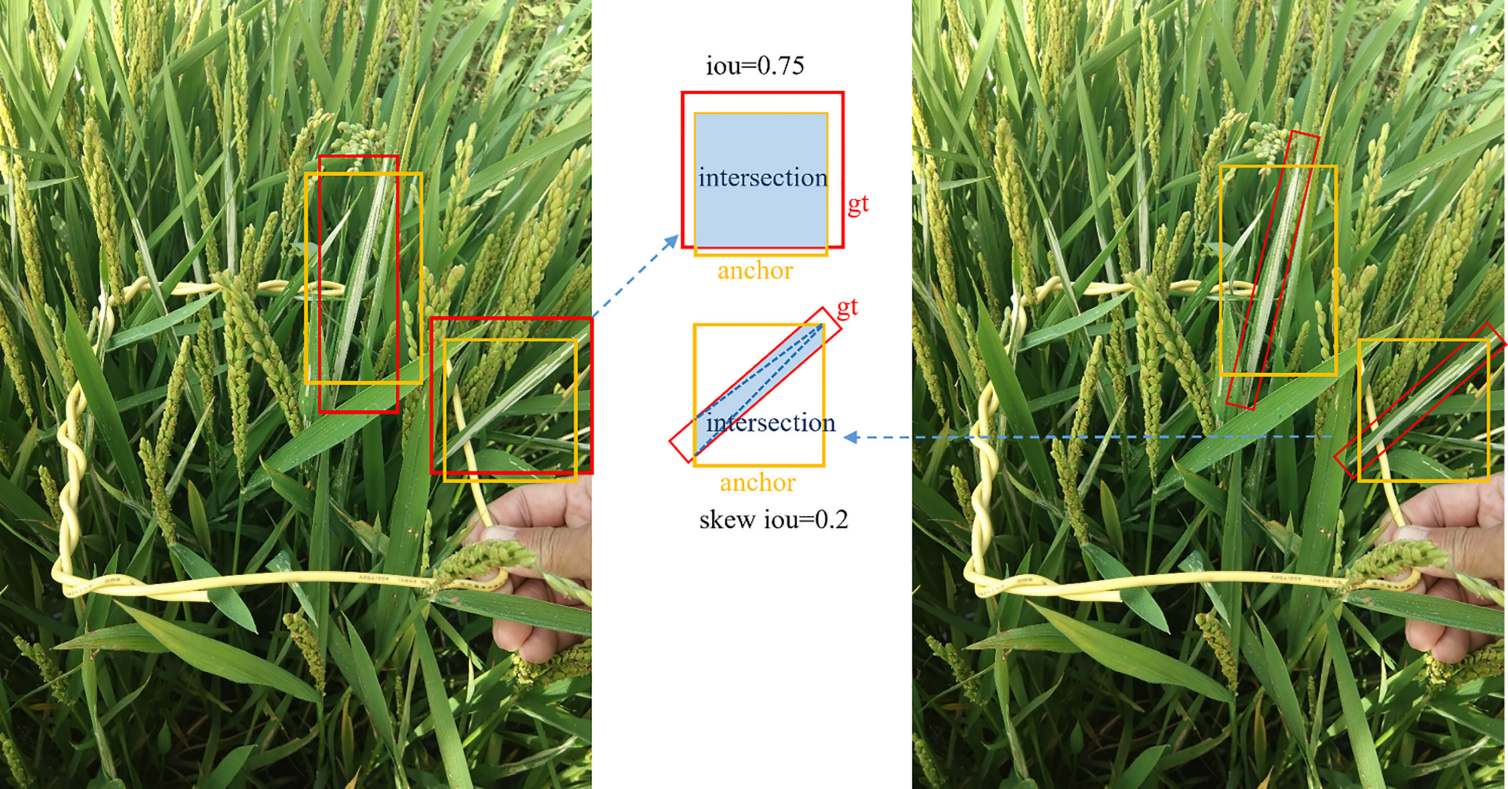
The skew IoU of the anchor (yellow) with the rotated ground truth (red RBBs in the right image) is significantly less than the IoU of the same anchor with the corresponding horizontal circumscribed rectangle (red HBBs in the left image). The blue area indicates the intersection between the anchor and the ground truth (gt).

**Figure 11:**
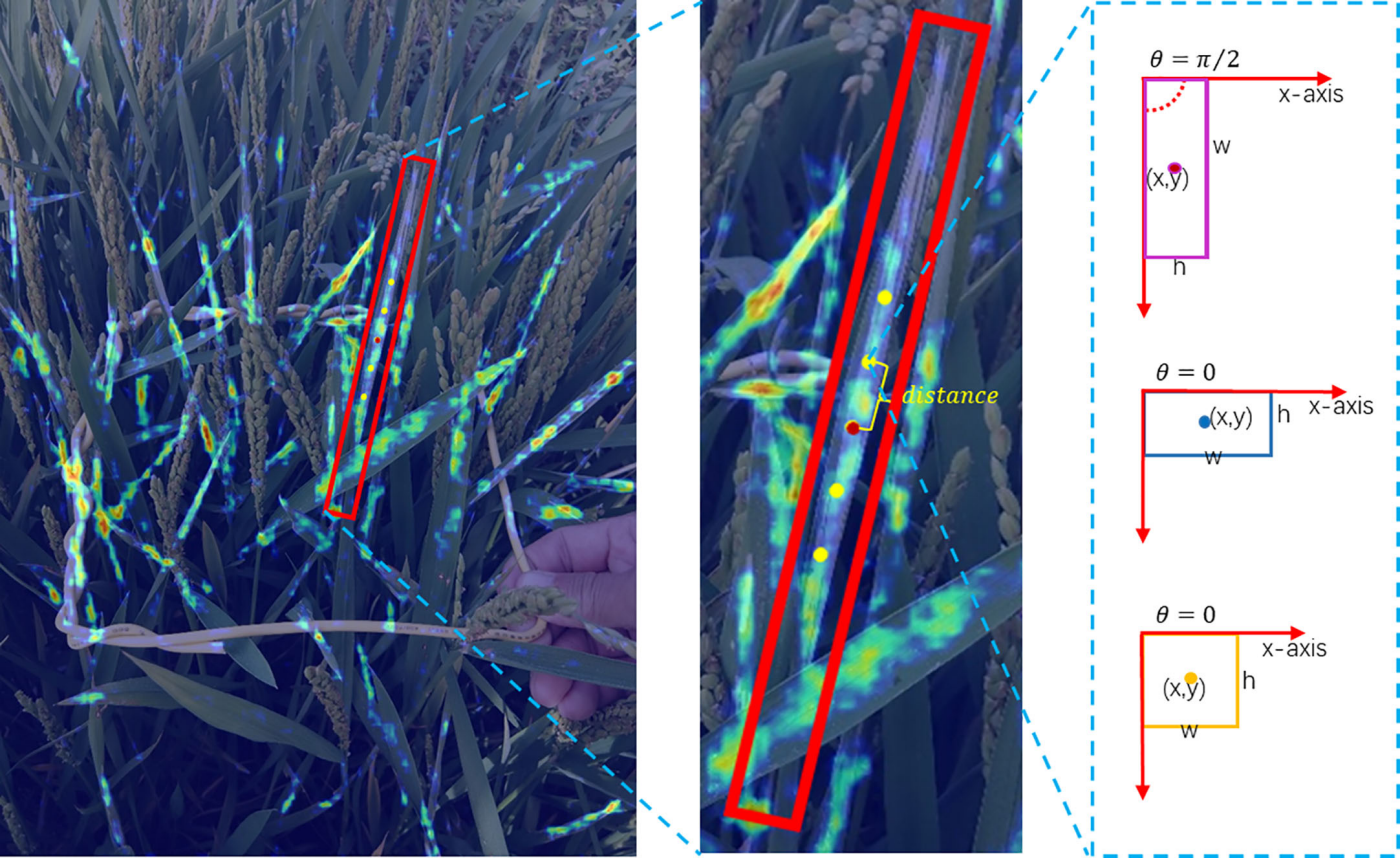
Anchor sample acquisition for a rotated instance on the P2-layer feature map.

During the training process, H2R-RPN adopts the adaptive training sample selection method (Zhang et al., 2020), which can find the optimal skew intersection over union (IoU) threshold for each rotated instance with horizontal anchors to separate positive and negative samples. The skew IoU definition is similar to the IoU and it can be calculated by the triangular dissection method based on intersection points (Ma et al., 2018). For a large range of aspect ratios and scales, it is challenging to set an appropriate skew IoU threshold to define positive and negative samples, as shown in **Figure 10**. For each ground truth, n anchors closest to its center point are obtained in each of m different feature layers for a total of m×n . **Figure 11** shows the anchor sample acquisition of an instance on the P2-layer feature map. The skew IoU threshold corresponding to this instance is calculated as mean(IoUs)+std(IoUs) . The IoUs are sets between these. anchors with the ground-truth, and std is the standard deviation operation of IoUs. This implementation dynamically fetches positive samples for each instance, ensuring that each instance possesses positive samples. Negative samples will be randomly selected from the remaining anchors. By default, N=256 training samples are taken for one image. The ratio of positive and negative samples is 1:1. The sample selection strategy is used only during the training phase, which does not increase the inference time.

N training samples are involved in the calculation of the classification loss $L_{cls}$ , and only $N_{p}$ positive samples are needed to perform the regression loss $L_{reg}$ , which is expressed as follows:


(1) $$L=\frac{1}{N}(\sum_{i=1}^{N}L_{cls}(p_{dt},p_{gt})+\sum_{i=1}^{N_{p}}L_{reg}(\mu_{\Delta gt},\mu_{\Delta dt}))$$

where $p_{dt}$ denotes the probability over classes, $p_{gt}$ denotes the class label ( $p_{gt}$ =1 for the positive sample, otherwise it is 0). $L_{cls}$ adopts the cross-entropy loss, $L_{reg}$ is calculated by the $smooth_{L_{1}}$ function that includes the regression of the center point coordinates (x,y) , the long and short edges (w,h) , and the angle θ :


(2) $$L_{reg}(\mu_{\Delta gt},\mu_{\Delta dt})=\sum_{i\in \{x,y,w,h,\theta \}}smooth_{L_{1}}(\mu_{\Delta gt}^{i}-\mu_{\Delta dt}^{i})$$

(3) $$smooth_{L_{1}}(x)=\{\begin{array}{c} 0.5x^{2},if\left|x\right|<1\\ \left|x\right|-0.5,otherwise \end{array}$$

where $\mu_{\Delta dt}^{i},i\in \{x,y,w,h,\theta \}$ is the regression result representing offsets between the detection box $i_{dt},i\in \{x,y,w,h,\theta \}$ and the predefined anchor $i_{a},i\in \{x,y,w,h,\theta \}$ , $\mu_{\Delta gt}^{i},i\in \{x,y,w,h,\theta \}$ denotes offsets between the ground truth $i_{gt},i\in \{x,y,w,h,\theta \}$ and the anchor. The specific calculations are as follows:


(4) $$\begin{array}{c} \mu_{\Delta gt}^{x}=(x_{gt}-x_{a})/w_{a},\mu_{\Delta dt}^{x}=(x_{dt}-x_{a})/w_{a}\\ \mu_{\Delta gt}^{y}=(y_{gt}-y_{a})/h_{a},\mu_{\Delta dt}^{y}=(y_{dt}-y_{a})/h_{a}\\ \mu_{\Delta gt}^{w}=\log(w_{gt}/w_{a}),\mu_{\Delta dt}^{w}=\log(w_{dt}/w_{a})\\ \mu_{\Delta gt}^{h}=\log(h_{gt}/h_{a}),\mu_{\Delta dt}^{h}=\log(h_{dt}/h_{a})\\ \mu_{\Delta gt}^{\theta }=\theta_{gt}-\theta_{a},\mu_{\Delta dt}^{\theta }=\theta_{dt}-\theta_{a} \end{array}$$

In the inference process, oriented proposal boxes are obtained by the above calculation between predicted results and anchors. Finally, the normalization operation $\theta_{dt}=(\mu_{\Delta dt}^{\theta }+\theta_{a}+\pi /4)\%\pi -\pi /4$ needs to be performed to limit the angle $\theta_{dt}$ range in [−π/4,3π/4) .

#### 2.2.3 Rotated-to-Rotated RCNN

The R2R-RCNN module comprises a rotated region of interest alignment (RRoIAlign) operation and a detection head. The R2R-RCNN relies on k×k×256 feature map regions aligned with rotated proposals for detection rather than a misaligned 256-dimensional feature vector. The rotated proposals obtained from the H2R-RPN stage are denoted as $\left(x_{p},y_{p},w_{p},h_{p},\theta \right)$ . The RRoIAlign operation projects the rotated proposals onto the feature map with the stride of s to obtain oriented-aligned feature regions denoted as $\left(x_{f},y_{f},w_{f},h_{f},\theta \right)$ as follows:


(5) $$\{\begin{array}{c} w_{f}=w_{p}/s,h_{f}=h_{p}/s\\ x_{f}=\left\lfloor x_{p}/s\right\rfloor ,y_{f}=\left\lfloor y_{p}/s\right\rfloor \end{array}$$

The rotated feature regions are then pooled into k×k grids with 256 channels to improve efficiency. For the index (m,n) grid in the c−th(0≤c<C) channel, the value is calculated as:


(6) $$F_{c}^{'}(m,n)=\sum_{\left(x,y)\in bin(m,n\right)}F_{c}(R_{\theta }(x,y))/l$$

(7) $$R_{\theta }(x,y)=\left(\begin{array}{cc} \cos\theta & -\sin\theta \\ \sin\theta & \cos\theta \end{array}\right)\left(\begin{array}{c} x-w_{f}/2\\ y-h_{f}/2 \end{array}\right)+\left(\begin{array}{c} x_{f}\\ y_{f} \end{array}\right)$$

where 0≤m,n<k , l represents the number of sampled points in one grid, $F_{c}(R_{\theta }(x,y))$ denotes the value in the c−th channel for the sampled point within each grid after the rotated operation $R_{\theta }(.)$ .

**Figure 12:**
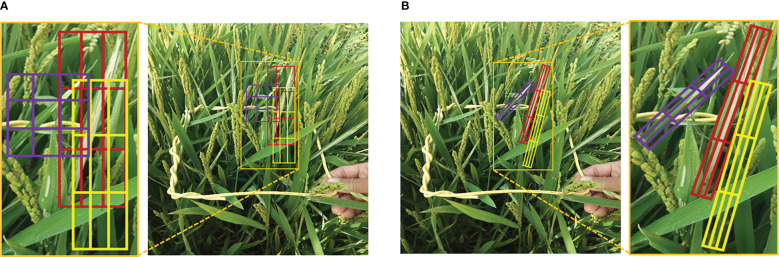
Feature alignment. **(A)** RoIAlign and **(B)** RRoIAlign. For simplicity, we exhibit the comparison results with 3×3 on the original image instead of feature maps.

The RRoIAlign operation is performed on arbitrary-oriented proposals, while RoIAlign is for horizontal proposals, as shown in **Figure 12**. We use the common setting 7×7 for the parameter k×k in the experiments. The sample values within each grid are calculated by bilinear interpolation. **Figure 12** reveals that RoIAlign introduces ambiguity, such as background or other instances, while RRoIAlign can focus more on discriminative features for rotated objects.

All proposals are transformed into fixed-size feature vectors of k×k×C by a RRoIAlign operation. Next, the feature vectors are fed into two cascaded fully-connected layers $Linear\left(d_{input},d_{output}\right)$ in the detection head, where $d_{input}$ and $d_{output}$ (by default $d_{output}=1024$ ) denote the dimensions of the input and output vectors. Finally, two sibling fully-connected layers, Linear(1024,2) and Linear(1024,5) , are used for the class probability and regression offset prediction, respectively.

## 3 Experiment

### 3.1 Experimental settings

The experimental platform uses Ubuntu 18.04 operating system with PyTorch 1.7.1, Python 3.7.10 and CUDA version 11.1. The GPU is NVIDIA TITAN RTX with 24G memory. The optimizer SGD (stochastic gradient descent) is adopted to train the models with 36 epochs and a batch size of 2. The learning rate is reduced at the 24th and 33rd epochs with a momentum of 0.9. The CMRD and CMHD datasets are utilized for rotated detection algorithms and horizontal detection algorithms based on the rotated object detection framework MMRotate (Zhou et al., 2022) and the generic object detection framework MMDetection (Chen et al., 2019). All the experiments use ResNet-50 (He et al., 2016) as the feature extraction backbone, and the FPN method (Lin et al., 2017a) as the feature fusion neck. The size of images is normalized to (1024,1024) . Other parameters keep the default settings.

### 3.2 Evaluation metrics

The evaluation metrics for rotated and horizontal detection are defined in the similar way, specifying an IoU threshold between the detection box and all labeled instances to determine whether the detection result is correct. Unlike horizontal detectors, rotation detectors are evaluated with skew IoU calculated by the triangulation method. For a given skew IoU threshold, a set of (P,R) values can be computed by setting different confidence score thresholds, where P(Precision) denotes the correct proportion of detection results and R (Recall) denotes the correctly detected proportion of labeled instances. The higher the confidence score threshold, the higher the P and the lower the R in general. The average precision (AP) is widely used to evaluate the overall performance of models by calculating the integral under the precision-recall curve, as in Formula 8. Considering that some *C.medinalis* damage symptoms are truncated and discontinuous, we also evaluate AP with skew intersection over foreground (IoF), which is the ratio of the intersection between the detection box and the instance to the detection box.


(8) $$\begin{array}{c} P=\frac{TP}{TP+FP}\times 100\%\\ R=\frac{TP}{TP+FN}\times 100\%\\ AP=\int_{0}^{1}P(R)dR \end{array}$$

where TP, FP and FN denote true positive, false positive and false negative, respectively.

### 3.3 Comparison between rotated detectors and horizontal detectors

The rotated detection algorithms produce oriented bounding boxes closely surrounding the damage symptoms. We compare the state-of-the-art rotated algorithms with horizontal detection algorithms, including our proposed method CMRD-Net. The results of AP with IoU=0.5 are shown in **Table 2**. It can be seen that our two-stage rotated CMRD-Net method achieves the highest AP with 73.7% among all algorithms listed. Within horizontal detection algorithms, the two-stage Doublehead-rcnn method achieves the best performance with 69.3% AP. The CMRD-Net and Roi-trans show higher AP than Doublehead-rcnn by 4.4% and 3.1%. Compared with the horizontal detection algorithms Faster-rcnn and Fcos, the rotated detection methods Rotated Faster-rcnn and Rotated Fcos obtain 0.8% and 1.9% improvements, respectively. The results show that compared with horizontal detection algorithms, our rotated detection algorithm provides precise localization while achieving better performance.

**Table 2:** Comparison between rotated detectors and horizontal detectors.

Mode	Method	AP (%)
Horizontal detection methods	Autoassign (Zhu et al., 2020)	63.5
	Fcos (Tian et al., 2019)	65.3
	Ddod (Chen et al., 2021)	64.9
	Dynamic Head (Dai et al., 2021)	67.6
	Libra-rcnn (Pang et al., 2019)	64.7
	Faster-rcnn (Ren et al., 2017)	68.6
	Cascade-rcnn (Cai and Vasconcelos, 2018)	69.1
	Doublehead-rcnn (Wu et al., 2020)	69.3
Rotated detection methods	Gliding Vertex (Xu et al., 2021)	66.1
	Rotated Fcos (Tian et al., 2019)	67.2
	Oriented Reppoints (Li et al., 2022)	70
	S2anet (Han et al., 2021)	70.7
	Rotated Faster-rcnn (Ren et al., 2017)	69.4
	Roi-trans (Ding et al., 2019)	72.4
	CMRD-Net	**73.7**

**Figure 13:**
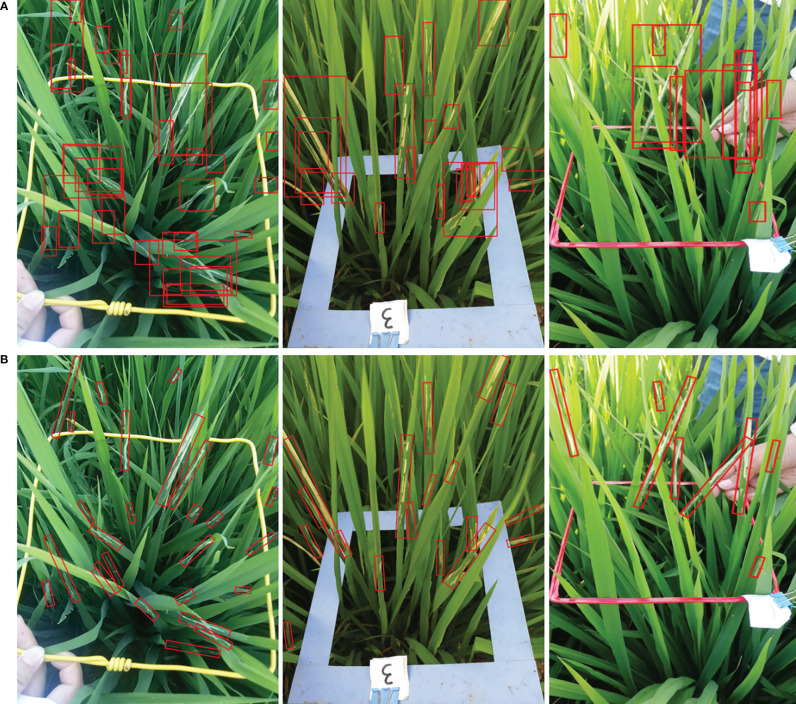
Examples of the comparison detection results of the horizontal detector with the rotated detector. The first row **(A)** is the detection results of Faster-rcnn and the second row **(B)** is the detection results of Rotated Faster-rcnn.

We visualize the detection results of Faster-rcnn and Rotated Faster-rcnn in **Figure 13**. The comparison figure reveals that the rotated detection boxes adhere more closely to the damaged areas and enhance the visualization ability of detection results. The horizontal detection algorithm Faster-rcnn can provide accurate horizontal bounding boxes when objects are sparsely distributed, but it is intractable to detect crossed and densely-distributed objects. The rotated detection methods present higher applicability in complex field conditions.

### 3.4 Comparison with other rotated detectors

We compare CMRD-Net with other state-of-the-art rotated detection algorithms. We calculate AP with a skew IoU threshold equal to 0.75 and 0.5, namely $AP_{75}$ and $AP_{50}$ . The same is for skew IoF. As shown in **Table 3**, the proposed CMRD-Net outperforms the other models. To be specific, CMRD-Net achieves the highest AP with 33.7%, 73.7%, 63.4% and 81.7% in four cases respectively, which is higher than the two-stage method Roi-trans by 1.2%, 1.3%, 2.1% and 5.3%. Among one-stage algorithms, Oriented Reppoints obtains the highest $AP_{75}(IoU)$ with 28.2.% and S2anet has the best performance in other three cases. Compared to the optimal one-stage algorithms, CMRD-Net obtains an improvement of 5.5%, 3.0%, 4.9% and 1.5% on $AP_{75}(IoU)$ , $AP_{50}(IoU)$ , $AP_{75}(IoF)$ and $AP_{50}(IoF)$ . We also calculate the parameter quantity (Params), the floating-point operations per second (FLOPs) and the frames per second (Fps) for all the algorithms. The FLOPs are related to the input size, which is uniformly fixed at 3×1024×1024 . For a fair comparison, the inference speed is the average speed with 5 times based on 1900 images in the testing set. Compared to one-stage algorithms, two-stage algorithms substitute feature point vectors with more feature regions corresponding to the region of interests for detection, resulting in higher Params and FLOPs in general. With an additional proposal refinement stage, the method Roi-trans is significantly inferior to CMRD-Net in terms of Params and detection speed. Our framework does not add extra parameters among two-stage algorithms and the detection speed is satisfactory for practical applications.

**Table 3:** Comparison with other rotated detectors.

Mode	Method	IoU		IoF		Params (M)	FLOPs (G)	Fps
		*AP*_75_ (%)	*AP*_50_ (%)	*AP*_75_ (%)	*AP*_50_ (%)			
One- Stage	R3det (Yang et al., 2021)	16.8	63.6	54	76.3	41.58	328.7	16.2
	Rotated Fcos (Tian et al., 2019)	24.8	67.2	56.7	77.9	31.89	206.2	24.9
	CFA (Guo et al., 2021)	24.7	67.5	49.2	74	36.6	194.24	21.3
	Rotated Atss (Zhang et al., 2020)	25.9	68.2	58.6	78.8	36.01	207.16	23.1
	Oriented Reppoints (Li et al., 2022)	28.2	70	51	74.7	36.6	194.24	21.4
	S2anet (Han et al., 2021)	24.4	70.7	58.5	80.2	38.54	196.21	19.6
Two- Stage	Gliding Vertex (Xu et al., 2021)	20.3	66.1	52.4	75.9	41.13	211.29	20
	Rotated Faster-rcnn (Ren et al., 2017)	25.5	69.4	58.6	78.8	41.12	211.28	20.3
	Roi-trans (Ding et al., 2019)	32.5	72.4	61.3	76.4	55.03	225.18	17
	CMRD-Net	**33.7**	**73.7**	**63.4**	**81.7**	41.12	211.35	19.4

**Figure 14:**
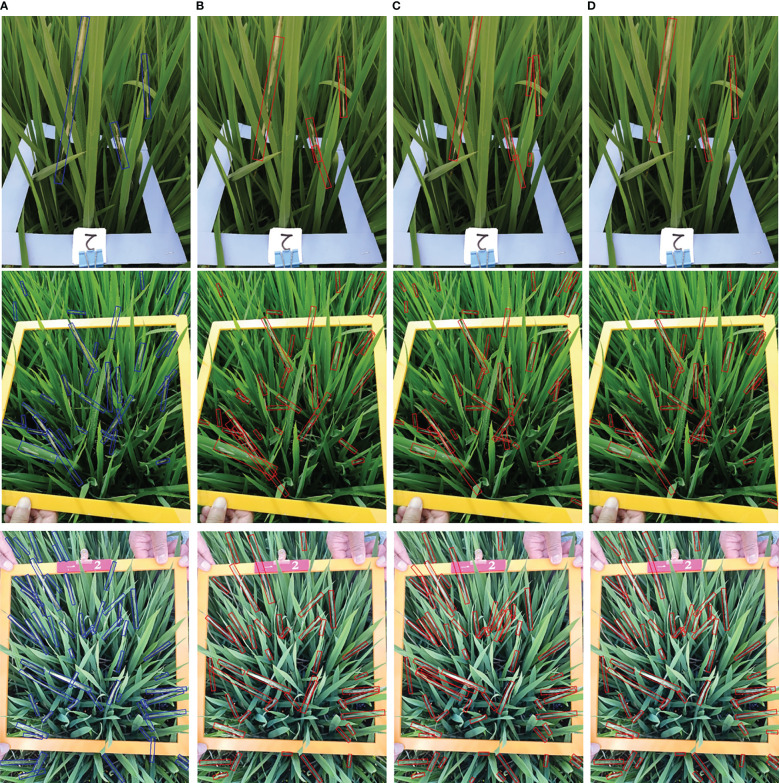
Examples of the comparison detection results with other rotated detection methods. The left-most column **(A)** shows Ground truth. The other three columns are detection results of the methods **(B)** Oriented Reppoints, **(C)** Rotated Faster-rcnn and **(D)** CMRD-Net.

**Figure 15:**
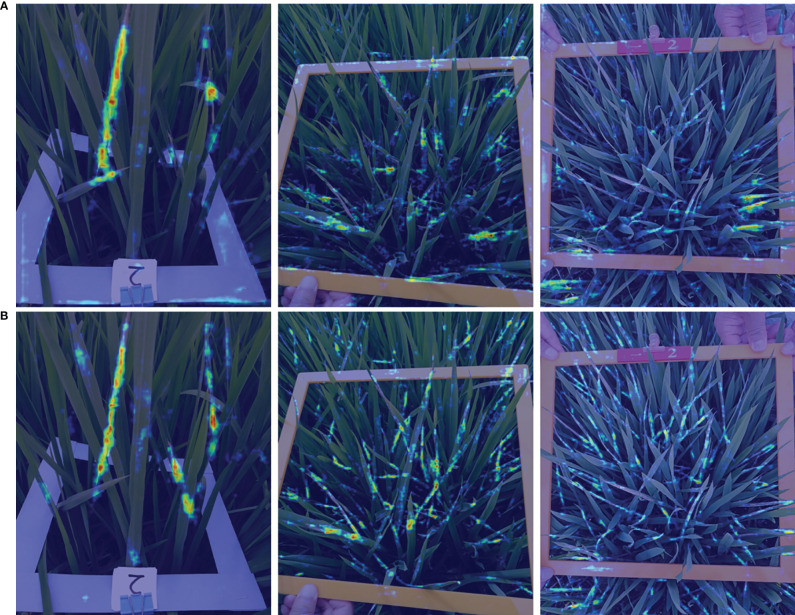
Visualization comparison of feature maps. The first row **(A)** shows the feature maps of Rotated Faster-rcnn(horizontal proposals-based) and the second row **(B)** shows the feature response maps of CMRD-Net(rotated proposals-based).

Examples of the comparison detection results with several other rotated detection methods are visualized in **Figure 14**. The left-most column shows the oriented annotations. The ground-truth image in the first row only has three separate instances. The second and third row illustrate images with crossed and densely packed objects. Comparatively, our proposed CMRD-Net obtains more accurate detection boxes compared to other methods for both sparse and dense distributions. **Figure 15** presents the feature maps of our method and Rotated Faster-rcnn. The primary difference between CMRD-Net and Rotated Faster-rcnn is that CMRD-Net generates rotated proposals in the region proposal network, whereas Rotated Faster-rcnn generates horizontal proposals. As can be seen from the comparison feature maps, our method obtains better discriminative response features indicating the importance of learning rotated proposals in the first stage network for two-stage algorithms.

### 3.5 Comparison in different scenes

To further validate the effectiveness of the proposed framework, we perform comparison experiments with other rotated algorithms on test subsets called “sparse”, “medium”, “dense” with different number of instances per image, and “sunlight” with illumination influence. We present AP with a skew IoU threshold equal to 0.5. **Table 4** shows that CMRD-Net achieves 76.8% in the sparse scene higher than Oriented Reppoints by 1.1%. Despite the fact that Roi-trans performs well on the other three scenes, CMRD-Net always obtains improvements than Roi-trans. Our method achieves the highest AP under different in-field scenes. It can also be seen that CMRD-Net reaches 65.4% and 63.9% in the dense and sunlight scene, which are not as well detected as the sparse scene 76.8% and the medium scene 73.8%, but are still superior to other state-of-the-art methods. The scenes with densely distributed objects and illumination influence need to be focused on and addressed in future work.

**Table 4:** Comparison with other rotated detectors in different scenes based on AP(%).

method	sparse	medium	dense	sunlight
R3det (Yang et al., 2021)	72.1	63.2	54.3	57.5
Rotated Fcos (Tian et al., 2019)	71.1	68	59.4	58.3
CFA (Guo et al., 2021)	71.3	69.3	56	58.9
Rotated Atss (Zhang et al., 2020)	74.2	66.8	59.8	59.5
Oriented Reppoints (Li et al., 2022)	75.7	70.8	55.7	61.2
S2anet (Han et al., 2021)	74.5	69.2	63.3	62.4
Gliding Vertex (Xu et al., 2021)	74.3	65.9	62.4	61.2
Rotated Faster-rcnn (Ren et al., 2017)	73.3	69.7	62.7	60.4
Roi-trans (Ding et al., 2019)	74.6	72.3	65.2	62.4
CMRD-Net	**76.8**	**73.8**	**65.9**	**64.3**

## 4 Discussion

As the *C.medinalis* pests conceal themselves in the rolled leaves, agricultural experts assess the pest occurrence level by estimating pest damage symptoms with visual observation during the field survey. To effectively control and prevent pest outbreaks, it is essential to detect pest damage symptoms automatically and precisely. Advances in deep learning techniques have boosted research into object recognition and detection for pest damage or disease symptoms (Lu et al., 2017; Rahman et al., 2020; Temniranrat et al., 2021; Debnath and Saha, 2022; Yang et al., 2022).

In complex field conditions, instance-level horizontal bounding box detectors based on deep learning are commonly used to locate pest damage or disease symptom regions (Zhou et al., 2019; Li et al., 2020; Yao et al., 2020; Pan et al., 2023). However, the oriented and densely-distributed object characteristics increase the difficulty of horizontal detection, making it challenging to detect damage symptom regions precisely. Comparatively, rotated bounding box detectors can provide more precise regions with orientation information and are better adapted to complex field environments. We propose a deep learning-based detection framework with rotated bounding box for in-field *C.medinalis* damage symptoms survey, called CMRD-Net.

The comparison performances between rotated and horizontal detectors are listed in **Table 2**. The rotated detection methods Roi-trans (72.4%) and our CMRD-Net (73.7%) achieve higher AP than the best performing horizontal detector Doublehead-rcnn (69.3%). Rotated Faster-rcnn and Rotated Fcos outperform Faster-rcnn and Fcos, respectively. **Figure 13** illustrates that the rotated detection boxes are more suitable for characterizing the oriented damage symptoms and favorable for inspecting their actual positions. Furthermore, our CMRD-Net is superior to other state-of-the-art rotated detection methods by four different evaluation indicators, as shown in **Table 3**. Meanwhile, CMRD-Net does not add additional parameters within two-stage algorithms, and the detection speed is satisfactory for real-world tasks. The comparative detection results in **Figure 14** show the excellent detection performance of our framework. **Figure 15** illustrates that CMRD-Net extracts more discriminative features, improving the feature representation capability based on rotated proposals. In addition, comparison experiments with other state-of-the-art rotated detection methods in different scenes further verify the effectiveness of the proposed framework, as shown in **Table 4**. The detection results under the four scenes show that we need to pay more attention to the scenes with densely-distributed objects and illumination effects.

## 5 Conclusion and future work

*C.medinalis* seriously affects the yield and quality of rice. The automatic detection method of its damage symptoms has become an urgent requirement and development trend for field investigation. Rice leaves grow in arbitrary-oriented directions under natural conditions, resulting in *C.medinalis* damage symptoms inclined, crossed and slender. We explore a two-stage rotated detection framework CMRD-Net to solve the above problems based on a newly constructed dataset named CMRD with oriented annotations. The extensive experimental results show that our proposed algorithm can achieve superior detection results among the state-of-the-art rotated detection methods. In addition, compared with horizontal detection methods, our rotated detection framework CMRD-Net obtains higher AP and locates the damage symptom regions more precisely. Our work provides novel insights into in-field *C.medinalis* investigation to take the initiative of pest control.

Despite the outstanding effect of our proposed rotated detection method for detecting *C.medinalis* damage symptoms in the field, there are still some limitations. The detection results are clearly separated when *C.medinalis* damage symptoms are heavily occluded. Occasionally, *C.medinalis* damage symptoms have curved shapes that do not facilitate rotated detection methods. In further research, we will consider the occlusion and bending, and design a unified detection framework that can handle the complex cases of inclination, occlusion, and bending simultaneously.

## Data availability statement

The original contributions presented in the study are included in the article/supplementary material. Further inquiries can be directed to the corresponding authors.

## Author contributions

TC: conceptualization, methodology, software, investigation, data collection and writing—original draft. HC: conceptualization and software. JD: formal analysis, writing—review and editing. WD and MZ: data annotation. RW, JD, and JZ: project supervision. RW: Funding acquisition. All authors contributed to the article and approved the submitted version.
